# Inter-regulation of IGFBP1 and FOXO3a unveils novel mechanism in ursolic acid-inhibited growth of hepatocellular carcinoma cells

**DOI:** 10.1186/s13046-016-0330-2

**Published:** 2016-03-31

**Authors:** Li Jun Yang, Qing Tang, Jingjing Wu, Yuqing Chen, Fang Zheng, Zhenhui Dai, Swei Sunny Hann

**Affiliations:** Laboratory of Tumor Biology and Target Therapy, The Second Clinical Medical Collage, University of Guangzhou Traditional Chinese Medicine, Guangzhou, Guangdong Province 510120 China; Department of Radiation Therapy, Guangdong Provincial Hospital of Chinese Medicine, The Second Clinical Medical Collage, University of Guangzhou Traditional Chinese Medicine, Guangzhou, Guangdong Province 510120 China; No. 55, Neihuan West Road, Higher Education Mega Center, Panyu District, Guangzhou, Guangdong Province 510006 P. R. China

**Keywords:** HCC, UA, p38 MAPK, IGFBP1, FOXO3a

## Abstract

**Background:**

Ursolic acid (UA), a natural pentacyclic triterpenoid, exerts anti-tumor effects in various cancer types including hepatocellular carcinoma (HCC). However, the molecular mechanisms underlying this remain largely unknown.

**Methods:**

Cell viability and cell cycle were examined by MTT and Flow cytometry assays. Western blot analysis was performed to measure the phosphorylation and protein expression of p38 MAPK, insulin-like growth factor (IGF) binding protein 1 (IGFBP1) and forkhead box O3A (FOXO3a). Quantitative real-time PCR (qRT-PCR) was used to examine the mRNA levels of IGFBP1 gene. Small interfering RNAs (siRNAs) method was used to knockdown IGFBP1 gene. Exogenous expressions of IGFBP1 and FOXO3a were carried out by transient transfection assays. IGFBP1 promoter activity was measured by Secrete-Pair™ Dual Luminescence Assay Kit. In vivo nude mice xenograft model and bioluminescent imaging system were used to confirm the findings in vitro.

**Results:**

We showed that UA stimulated phosphorylation of p38 MAPK. In addition, UA increased the protein, mRNA levels, and promoter activity of IGFBP1, which was abrogated by the specific inhibitor of p38 MAPK (SB203580). Intriguingly, we showed that UA increased the expression of FOXO3a and that overexpressed FOXO3a enhanced phosphorylation of p38 MAPK, all of which were not observed in cells silencing of endogenous IGFBP1 gene. Moreover, exogenous expressed IGFBP1 strengthened UA-induced phosphorylation of p38 MAPK and FOXO3a protein expression, and more importantly, restored the effect of UA-inhibited growth in cells silencing of endogenous IGFBP1 gene. Consistent with these, UA suppressed tumor growth and increased phosphorylation of p38 MAPK, protein expressions of IGFBP1 and FOXO3a in vivo.

**Conclusion:**

Collectively, our results show that UA inhibits growth of HCC cells through p38 MAPK-mediated induction of IGFBP1 and FOXO3a expression. The interactions between IGFBP1 and FOXO3a, and feedback regulatory loop of p38 MAPK by IGFBP1 and FOXO3a resulting in reciprocal pathways, contribute to the overall effects of UA. This in vitro and in vivo study corroborates a potential novel mechanism by which UA controls HCC growth and implies that the rational targeting IGFBP1 and FOXO3a can be potential for the therapeutic strategy against HCC.

## Background

Hepatocellular carcinoma (HCC) is the third leading cause of cancer-related deaths globally characterized by high malignancy, aggressive progression, clinical difficulty and limited therapeutic options, resulting in poor prognosis and remaining a significant clinical challenge [1–4]. Usually, HCC shows high mortality even after treatments, such as chemotherapy and surgical resection, microwave ablation, trans-arterial chemoembolization, targeted therapy and liver transplantation [5]. Furthermore, the management of patients with HCC is complex due to the complicated molecular pathogenesis, incurable advanced stages, and adverse responses from available anti-HCC drugs [6, 7]. Thus, it is necessary to develop new effective therapeutic strategies to improve the quality of life and survival of patients with HCC. There is currently increasing interest in Traditional Chinese Medicine (TCM) herbal mixtures and its components, which have been used to treat malignant tumors including HCC with potentially beneficial outcomes [8–10]. However, the detailed mechanisms by which TCM and extracted components suppress growth of cancers including HCC hitherto remain to be understood.

Ursolic acid (UA), a natural pentacyclic triterpenoid carboxylic acid obtained from TCM herbs and edible plants, exhibits potential anticancer effects through multiple mechanisms in various human cancers including HCC [10–14]. Our previous studies have showed that UA inhibited growth of HCC cells through AMP-activated protein kinase alpha (AMPKα)-mediated inhibition of transcription factor Sp1 and epigenetic regulator DNA (cytosine-5-)-methyltransferase 1 (DNMT1) [10]. Others found that ursolic acid induced apoptosis in HepG2 HCC cells via activation/phosphorylation of AMPK and glycogen synthase kinase 3 beta (GSK3β) [15]. However, the precise mechanisms of UA in the control of HCC growth remain to be determined.

Insulin-like growth factor (IGF) binding protein 1 (IGFBP1), a pivotal protein of the IGF system, has been shown to be implicated in many cellular functions including proliferation, development, apoptosis, DNA damage repair, and tumor growth through IGF-dependent and -independent mechanisms [16–18]. Early report showed that inhibition of IGF receptor 1 function by IGFBP1 inhibited breast cancer cell growth [19]. Metformin, an activator of AMPK, a central metabolic regulator, was found to increase IGFBP1 expression, thereby inhibiting endometrial cancer cell proliferation [20]. The role of IGFBP1 in HCC has been reported, demonstrating that IGFBP1 inhibited the invasion and metastasis of HCC cells, and this could be considered as an important marker for the prognosis of HCC [21, 22]. Nevertheless, the insight true role of IGFBP1 in cancer cell biology, especially in growth and progression of HCC, still remains controversial.

Human forkhead box class O (FOXO) transcription factors implicated in a wide variety of cellular activities, such as differentiation, cell cycle, metabolism, stress resistance, mitogenic signaling, and tumor suppression [23]. Among four members (FOXO1, FOXO3a, FOXO4, and FOXO6), FOXO3a has been shown as a critical protein involving in proliferation, cell cycle arrest, apoptosis, differentiation, and metabolism [24–27]. FOXO3a acted as tumor suppressors and reduced expression of FOXO3a was associated with poor prognosis in gastric cancer patients [28]. On the contrary, exogenous expression of FOXO3a suppressed cancer cell growth through regulating downstream signaling molecules [25, 26, 29]. These results indicated a tumor suppressor role of FOXO3a, which could be a potential target for the treatment of cancers.

In this study, we further explore the potential mechanism by which UA controls growth of HCC cells. Our results in vitro and in vivo indicated that UA inhibited growth of HCC cells through p38 mitogen-activated protein kinase (MAPK)-mediated induction of IGFBP1 and FOXO3a expressions.

## Methods

### Reagents and cell culture

Monoclonal antibodies specific for total p38 MAPK and the phosphor-form (Thr180/Tyr182) were purchased from Cell Signaling Technology Inc. (Beverly, MA, USA). The IGFBP1 and FOXO3a antibodies were obtained from Epitomics (Burlingame, CA, USA). SB203580 was purchased from Merck Millipore (Darmstadt, Germany). MTT powder was purchased from Sigma Aldrich (St. Louis, MO, USA). Ursolic acid was purchased from Chengdu Must Bio-technology Company (Chengdu, Sichuan, China). Lipofectamine 3000 reagent was purchased from Invitrogen (Carlsbad, CA, USA). The drugs were freshly diluted to the final concentration in culture medium before experiment. Human HCC cell lines HepG2, Bel-7402, QGY-7703, HMCC97L and HMCC97H were obtained from the Cell Line Bank at the Laboratory Animal Center of Sun Yat-sen University (Guangzhou, China) and the Chinese Academy of Sciences Cell Bank of Type Culture Collection (Shanghai, China). The cells were cultured at 37 °C in a humidified atmosphere containing 5 % CO_2_. The culture medium consisted of RPMI1640 medium (GIBCO, Shanghai, China) supplemented with 10 % (v/v) heat-inactivated fetal bovine serum (Thermo Fisher Scientific Inc, MA, USA), 100 μg/ml streptomycin and 100 U/mL penicillin. When cells reached 70 % confluence, they were digested with 0.25 % trypsin for the following experiments.

### Cell viability assay

Cell viability was measured using the 3-(4, 5-dimethylthiazol-2-yl)-2, 5-diphenyltetrazolium bromide (MTT) assay and described previously [26, 30]. Briefly, HCC cells were harvested and seeded into a 96-well microtiterplate. The cells (5 × 10^3^ cells/well) were treated with increasing concentrations of UA for up to 72 h. After incubation, 10 μL MTT solution (5 g/L) was added to each well and HCC cells were incubated at 37 °C for an additional 4 h. Supernatant was removed, then 100 μL solvent dimethyl sulfoxide (DMSO) was added to each well and oscillated for 5 min. Absorbance at 570 nm was determined by ELISA reader (Perkin Elmer, Victor X5, Waltham, MA, USA). Cell viability (% of control) was calculated as (absorbance of test sample/absorbance of control) × 100 %.

### Cell cycle analysis

This procedure was reported previously [31]. In brief, HCC cells were cultured in 6-well plates and treated with increased doses of UA for 24 h. Afterwards, the cells were harvested, and resuspended in 500 μL of cold PBS for 2 h at 4 °C. Following washes, the fixed cells were incubated in 1 mL of 0.1 % sodium citrate containing propidium iodide (PI) 0.05 mg and 50 μg RNase for 30 min at room temperature (RT), subjected to FACSCalibur flow cytometric analysis (FC500, Beckman Coulter, FL, USA). The proportion of cells within the G0/G1, S and G2/M phases was analyzed using the MultiCycle AV DNA Analysis software (Phoenix Flow Systems).

### Treatment with FOXO3a and IGFBP1 siRNAs

The detailed procedure was reported previously [26]. For the transfection procedure, cells were seeded in 6-well or 96-well culture plates in RPMI 1640 medium containing 10 % FBS (no antibodies), grown to 60 % confluence, and FOXO3a, IGFBP1 and control siRNAs (up to 50 nM) purchased from Life Technologies (Carlsbad, CA, USA) were transfected using the Lipofectamine RNAiMAX Transfection Reagent (Grand Island, NY, USA) according to the manufacturer’s instructions. After culturing for up to 24 h, the cells were washed and resuspended in fresh media in the presence or absence of UA for an additional 24 h for all other experiments.

### Transient transfection assays

The detailed procedure was reported previously [32]. In brief, HCC cells were seeded at a density of 5 x10^5^ cells/well in 6-well dishes and grown to 60 % confluence. For each well, 2 μg of the desired N1-GFP or FOXO3a-GFP plasmid DNA, kindly provided by Frank M. J. Jacobs (Rudolf Magnus Institute of Neuroscience, Department of Pharmacology and Anatomy, University Medical Center, Utrecht, Netherlands) and was reported previously [33] and the control (pCMV-6) or IGFBP1 expression vectors (IGFBP1-pCMV6) purchased from OriGene Technologies, Inc. (Rockville, MD, USA) at a final concentration of 2 μg/mL were transfected into the cells using the lipofectamine 3000 reagent according to the manufacturer’s instructions for up to 24 h, followed by treating with UA for an additional 24 h. In separated experiment, cell were transfected with pEZX-PG04-IGFBP1 promoter construct linked Gaussia luciferase (GLuc) gene and secreted alkaline phosphatase (SEAP) internal control obtained from GeneCopoeia, Inc. (GeneCopoeia, Inc., Rockville, MD, USA). The preparation of cell extracts and measurement of luciferase activities were determined using the Secrete-Pair Dual Luminescence Assay Kit (GeneCopoeia, Inc., Rockville, MD, USA). Gaussia luciferase activity was normalized with SEAP within each sample.

### Quantitative real-time PCR

A quantitative real-time PCR (qRT-PCR) assay was used to detect IGFBP1. The primers used in this study were designed as follows: IGFBP1 forward 5′- TCACAGCAGACAGTGTGAGAC −3′; reverse 5′- CCCAGGGATCCTCTTCCCAT −3′; GAPDH forward 5′- AAGCCTGCCGGTGACTAAC −3′; reverse 5′- GCGCCCAATACGACCAAATC −3′. In brief, qRT-PCR was performed in a 20 μL mixture containing 2 μL of the cDNA preparation, 10 μL 2X SYBR Green Premix ExTaq (Takara), and 10 μM primer on an ABI 7500 Real-Time PCR System (Applied Biosystems, Grand Island, NY, USA). The PCR conditions were as follows: 10 min at 95 °C, followed by 40 cycles of 15 s at 95 °C, and 1 min at 60 °C. Each sample was tested in triplicate. Threshold values were determined for each sample/primer pair, the average and standard errors were calculated.

### Western blot

The detailed procedure was reported previously [26, 30]. Briefly, cell lysates containing equal amounts of protein concentration were separated on 10 % SDS polyacrylamide gels. Membranes (Millipore, Shanghai, China) were incubated with antibodies against p38 MAPK, p-p38 MAPK, FOXO3a and IGFBP1 (1:1000). The membranes were washed and incubated with a secondary antibody raised against rabbit IgG conjugated to horseradish peroxidase (Cell Signaling, Shanghai, China). The membranes were washed again and transferred to freshly made ECL solution (Immobilon Western; Millpore, Shanghai, China), followed by observing the signals under the Molecular Imager ChemiDoc XRS Gel Imagine System (BioRad, Hercules, CA, USA) and documenting the results.

### Xenograft tumors and bioluminescent imaging

In order to explore the effects and mechanisms of UA on tumor growth in vivo, a xenografted nude mouse model of HCC cells was established. Animal experiments were approved by Institutional Animal Care and Use Committee Animal Care of Guangdong Provincial Hospital of Chinese Medicine (the Ethics Approval Number 2014012). A total of 36 female nude mice (eight-week-old) obtained from Guangdong Provincial Research Center for Laboratory Animal Medicine (Foshan, Guangdong, China), were obtained and maintained at the Animal Center of Guangdong Provincial Hospital of Chinese Medicine in a specific pathogen-free environment with food and water provided. HepG2 cells carrying luciferase report gene (HepG2-Luc, obtained from the Guangzhou Land Technology Co., Guangzhou, China) (1x10^6^ cells) in 100 μL PBS were injected subcutaneously in nude mice. Xenografts were allowed to grow for over one week when the initial measurement was made with calipers and with bioluminescence imaging (BLI) using the IVIS-200 Imaging System (Xenogen Corporation, Berkeley, CA). The mice were randomly divided into control, low (25 g/kg), and high doses (50 g/kg) of UA treatment groups, which based on other studies [34–36]. The UA was given via gavages daily for up to 30 days (*n* = 12/group).

For bioluminescence imaging (BLI) procedure, the mice were anesthetized by inhalation of 2 % isoflurane at the end of experiment. Each set of mice were injected subcutaneously (dorsal midline) with 150 mg/kg D-luciferin (Xenogen; PerkinElmer, Waltham, MA, USA) in approximately 200 μL. Imaging and quantification of signals (photons/sec) were controlled by the acquisition and analysis software living image (version 1; Xenogen). Tumor volume measurements were calculated using the formula for an oblong sphere: volume = (width^2^ × length). The body weights of the mice were measured once a week. All mice were sacrificed on 30 days after each treatment using CO_2_ for euthanasia. The corresponding xenografted tumors were processed for detecting the phosphorylation of p38 MAPK, IGFBP1 and FOXO3a proteins by Western blot.

### Statistical analysis

The data are reported as means ± SD of at least three repeated experiments in triplicate measures. Differences between groups were assessed by one-way ANOVA and significance of difference between particular treatment groups was analyzed using Dunnett’s multiple comparison tests (GraphPadPrism5.0 software, LaJolla, CA). The results were presented relative to the control. Asterisks showed in the figures indicate significant differences of experimental groups in comparison with the corresponding control condition. A probability (*p*) value of <0.05 was considered to be significant.

## Results

### UA inhibited growth of HCC cells in the dose-dependent fashion

**Fig. 1:**
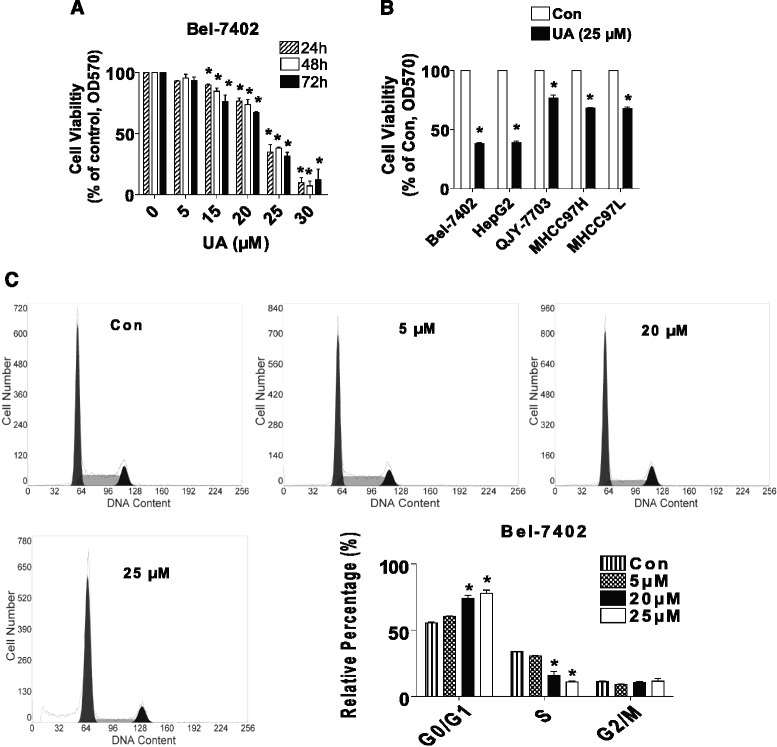
UA inhibited growth of HCC cells in the dose-dependent fashion. **a**, Bel-7402 cells were treated with increased concentrations of UA for up to 72 h to examine the cell viability. **b**, HCC cell lines indicated were treated with UA (25 μM) for 48 h. Afterwards, the cell viability was determined using the MTT assay as described in the Materials and Methods Section and was expressed as percentage of control in the mean ± SD of three separate experiments. *Indicates significant difference as compared to the untreated control group (*P* < 0.05). **c**, Bel-7402 cells were stimulated with different concentrations (e.g., 5, 20, 25 μM) of UA for up to 24 h. The cells were collected and processed for analysis of cell cycle distribution. Cell cycle was analyzed by flow cytometry after propidium iodide (PI) staining, and the percentages of the cell population in each phase (G0/G1, S and G2/M) of cell cycle were analyzed by Multicycle AV DNA Analysis Software. Data are expressed as a percentage of total cells. Values are given as the mean ± SD, from 3 independent experiments performed in triplicate. *Represents *P* < 0.05 versus control group

We previously showed that UA suppressed growth of HepG2 HCC cells [10]. In order to prove if this was the case in other HCC cell types, we further tested the effect of UA on the proliferation in other HCC cell lines. As shown in Fig. 1a, UA inhibited proliferation of Bel-7402 HCC cells in the dose-dependent manner with a significant inhibition observed at 25–30 μM ranges of UA treatment starting at 24 and up to 72 h as determined by MTT assays. The IC50 was 23.067 μM. Similar results were also observed in other HCC cell lines (Fig. 1b). We next performed the cell cycle experiment. As expected, compared with the untreated control cells, UA significant increased the proportion of cells at G0/G1 phase (>19 %), while the proportion of cells at S phases were reduced (Fig. 1c) suggesting that UA induced cell cycle arrest in G0/G1 phase in Bel-7402 cells.

### UA induced phosphorylation of p38 MAPK

**Fig. 2:**
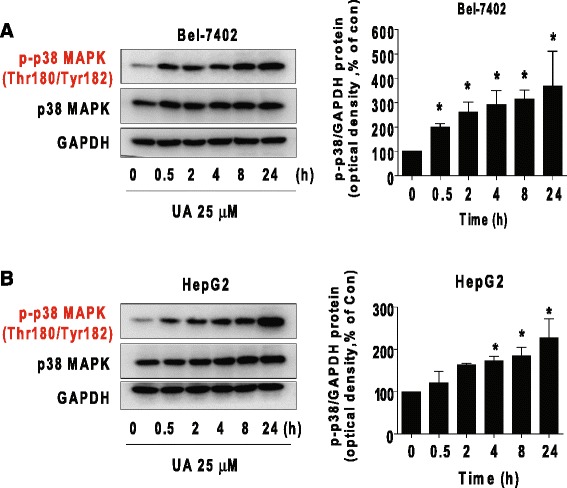
UA induced phosphorylation of p38 MAPK. **a**-**b**, Bel-7402 (**a**) and HepG2 (**b**) cells were exposed to UA (25 μM) for 24 h, followed by measuring the phosphorylation and protein expression of p38 MAPK by Western blot. The bar graphs represent the mean ± SD of p-p38 MAPK/GAPDH of three independent experiments. *Indicates significant difference as compared to the zero time group (*P* < 0.05)

We then explore the signaling pathway that may mediate the overall response of UA. P38 MAPK signaling pathway have been shown to be involved in growth, differentiation and progression of cancer [37]. Herein, we showed that UA increased phosphorylation of p38 MAPK, while it had little effect on total p38 MAPK protein in the time-dependent manner in Bel-7402 and HepG2 cells (Fig. 2a–b).

### UA induced the expression of protein, mRNA, and promoter activity of IGFBP1 through p38 MAPK signaling

**Fig. 3:**
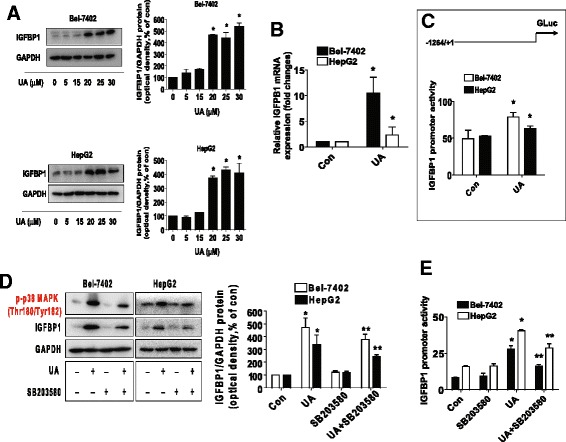
UA induced the protein, mRNA expression, and promoter activity of IGFBP1, which were blocked by SB203580. **a**-**b**, HepG2 and Bel-7402 cells were exposed to increased concentrations of UA or UA (25 μM) for 24 h. Afterwards, the expression of IGFBP1 protein (**a**) and mRNA (**b**) were detected by Western blot and qRT-PCR methods as described in the Materials and Methods section. *Indicates significant difference as compared to the untreated control group (*P* < 0.05) **c**, Bel-7402 and HepG2 cells were tranfected with wild type human IGFBP1 promoter reporter construct ligated to luciferase reporter gene and internal control secreted alkaline phosphatase (SEAP) for 24 h, followed by treating with UA (25 μM) for an additional 24 h. Afterwards, the IGFBP1 promoter activity were detected by the Secrete-Pair Dual Luminescence Assay Kit. **d**, HepG2 and Bel-7402 cells were treated with SB203580 (10 μM) for 2 h before exposure of the cells to UA (25 μM) for an additional 24 h. Afterwards, the expression of IGFBP1 protein and phosphorylation of p38 MAPK were detected by Western blot. The bars represent the mean ± SD of at least three independent experiments for each condition. *Indicates significant difference as compared to the untreated control group (*P* < 0.05); **Indicates significance of combination treatment as compared with UA alone (*P* < 0.05). **e**, Cellular protein was isolated from Bel-7402 and HepG2 cells cultured for 2 h in the presence or absence of SB203580 (10 μM) before transfection with control or above IGFBP1 constructs and exposing the cells to UA (25 μM) for an additional 24 h. Afterwards, the IGFBP1 promoter activity were detected by the Secrete-Pair Dual Luminescence Assay Kit. The bar graphs represent the mean ± SD of three independent experiments. *Indicates significant difference as compared to the untreated control group (*P* < 0.05); **Indicates significance of combination treatment as compared with UA alone (*P* < 0.05)

We next characterized the potential mechanism underlying this effect. Studies demonstrated that expression of IGFBP1 was associated with p38 MAPK signaling and involved in cancer cell growth [38]. In this study, we found that UA increased the protein expression of IGFBP1 in a dose-dependent fashion, with a significant induction seen at 20–30 μM ranges of UA treatment (Fig. 3a). UA also induced mRNA levels and promoter activity of IGFBP1 gene as determined by quantitative real-time PCR (qRT-PCR) and Dual Luminescence Assays, respectively (Fig. 3b–c). Interestingly, a specific inhibitor of p38 MAPK (SB203580) abolished the effect of UA on protein expression and promoter activity of IGFBP1 in Bel-7402 and HepG2 cells (Fig. 3d–e). Note that SB203580 inhibited the phosphorylation of p38 MAPK demonstrating the feasibility of this experiment (Fig. 3d–e). The findings above suggested that induction of IGFBP1 expression by UA was through the activation of p38 MAPK signaling pathway.

### UA increased FOXO3a protein expression through activation of p38 MAPK and expression of IGFBP1

**Fig. 4:**
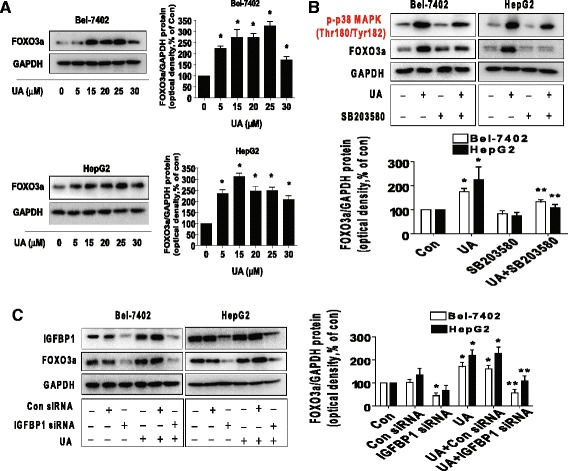
UA increased FOXO3a protein expression through activation of p38 MAPK and expression of IGFBP1. **a**, Bel-7402 and HepG2 cells were exposed to increased concentrations of UA for 24 h. Afterwards, the expression of FOXO3a protein was detected by Western blot. **b**, Bel-7402 and HepG2 cells were treated with SB203580 (10 μM) for 2 h before exposure of the cells to UA (25 μM) for an additional 24 h. Afterwards, the expression of FOXO3a protein and phoisphorylation of p38 MAPK were detected by Western blot. **c**, Bel-7402 and HepG2 cells were transfected with control or IGFBP1 siRNAs (50 nM each) for 24 h prior to exposure of the cells to UA (25 μM) for an additional 24 h. Afterwards, FOXO3a and IGFBP1 protein expressions were determined by Western blot, The bars represent the mean ± SD of at least three independent experiments for each condition. *Indicates significant difference as compared to the untreated control group (*P* < 0.05); **Indicates significance of combination treatment as compared with UA alone (*P* < 0.05)

Furthermore, we examined the potential downstream effectors of IGFBP1. Previous studies demonstrated the links of IGFBP1 and FOXO3a [39, 40]. For this reason we explored the role of FOXO3a. We found that UA also induced protein expression of FOXO3a in the dose-dependent fashion in Bel-7402 and HepG2 cells (Fig. 4a). And, this was eliminated in the presence of SB203580 (Fig. 4b) and in cells silencing of endogenous IGFBP1 gene using small interfering RNAs (siRNAs) (Fig. 4c). These results indicated the roles of p38 MAPK activation and expression of IGFBP1 in this process.

### Silencing of FOXO3a overcame UA-inhibited cell growth and exogenous expressed FOXO3a enhanced UA-induced phosphorylation of p38 MAPK through IGFBP1

**Fig. 5:**
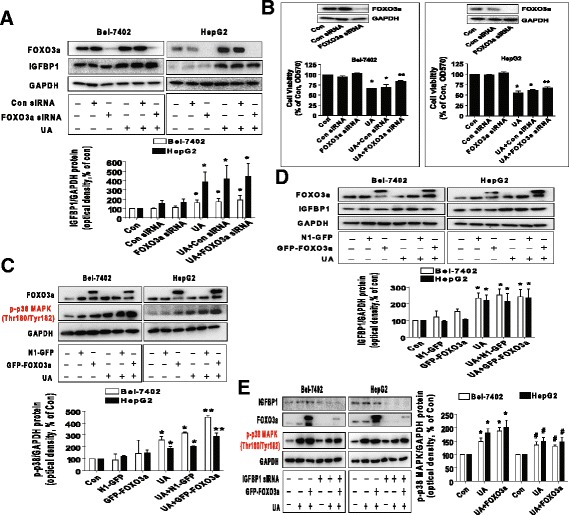
Silencing of FOXO3a overcame UA-induced cell growth inhibition and exogenous expressed FOXO3a enhanced UA-induced phosphorylation of p38 MAPK through IGFBP1. **a**, Bel-7402 and HepG2 cells were transfected with control and FOXO3a siRNAs for 24 h before exposing the cells to UA (25 μM) for an additional 24 h. Afterwards, FOXO3a and IGFBP1 protein expressions were determined by Western blot. **b**, Bel-7402 and HepG2 cells were transfected with control or FOXO3a siRNAs (up to 50 nM each) for 24 h prior to exposure of the cells to UA (25 μM) for an additional 24 h. Afterwards, FOXO3a protein expression and cell viability were determined by Western blot and MTT assays. Insert represents the protein expression of FOXO3a. **c**-**d,** Bel-7402 and HepG2 cells were transfected with control and FOXO3a overexpression vectors for 24 h before exposing the cells to UA (25 μM) for an additional 2 and 24 h, respectively. Afterwards, the protein levels of FOXO3a and p-p38 MAPK, and IGFBP1 protein expression were examined by Western blot. **e**, Bel-7402 and HepG2 cells silenced of IGFBP1 by siRNA previously were transfected with control and FOXO3a overexpression vector for 24 h before exposing the cells to UA (25 μM) for an additional 2 and 24 h, respectively. Afterwards, IGFBP1, FOXO3a protein and phosphorylation of p38 MAPK were determined by Western blot. Values in bar graphs were given as the mean ± SD from three independent experiments performed in triplicate. *Indicates significant difference as compared to the untreated control group (*P* < 0.05). **Indicates significant difference from UA treated alone (*P* < 0.01). ^#^Indicates significant difference as compared to the IGFBP1 siRNA alone group (*P* < 0.05)

In order to understand the potential role of FOXO3a, we had knockdown FOXO3a gene using siRNA method. As shown in Fig. 5a–b, silencing of FOXO3a had no effect on UA-induced IGFBP1 protein expression (A); however, it abolished the inhibitory effect of UA on cell growth in Bel-7402 and HepG2 cells (B). Moreover, we found that exogenous expressed FOXO3a enhanced UA-induced phosphorylation of p38 MAPK, but had little effect on expression of IGFBP1 protein (Fig. 5c–d). Intriguingly, the feedback regulation of p38 MAPK by FOXO3a was eliminated in cells silencing of endogenous IGFBP1 gene (Fig. 5e). Together, these findings above implied the upstream role of IGFBP1, and the expression of IGFBP1 was required for the complicated feedback regulation loops of p38 MAPK by FOXO3a, which resulted in the overall effects of UA in this process.

### Overexpression of IGFBP1 enhanced the effect of UA on FOXO3a expression and phosphorylation of p38 MAPK, and restored UA-inhibited growth in cells silencing of endogenous IGFBP1 gene

**Fig. 6:**
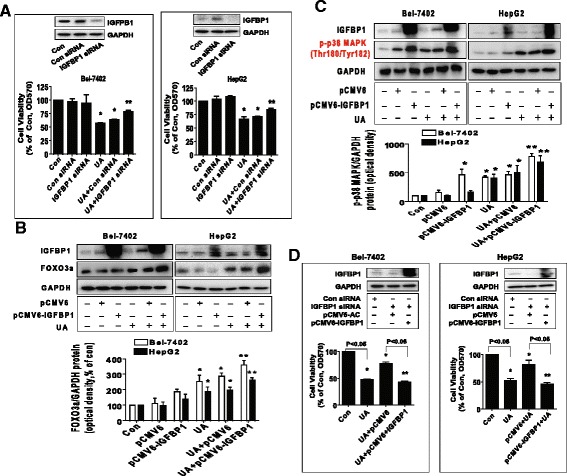
Overexpression of IGFBP1 enhanced the effect of UA on FOXO3a expression and phosphorylation of p38 MAPK, and restored UA-inhibited cell growth in cells silencing of endogenous IGFBP1 gene. **a**, Bel-7402 and HepG2 cells were transfected with control or IGFBP1 siRNAs (50 nM each) for 24 h prior to exposure of the cells to UA (25 μM) for an additional 24 h. Afterwards, IGFBP1 protein expression and cell viability were determined by Western blot and MTT assays. **b**-**c**, Bel-7402 and HepG2 cells were transfected with control and IGFBP1 overexpression vectors for 24 h before exposing the cells to UA (25 μM) for an additional 2 and 24 h, respectively. Afterwards, IGFBP1, FOXO3a protein levels and phosphorylation of p38 MAPK were determined by Western blot. **d**, Bel-7402 and HepG2 cells silenced of IGFBP1 by siRNA previously were transfected with control and IGFBP1 overexpression vectors for 24 h before exposing the cells to UA (25 μM) for an additional 24 h. Afterwards, IGFBP1 protein expressions and cell viability were determined by Western blot and MTT assays. Values in bar graphs were given as the mean ± SD from three independent experiments performed in triplicate. *Indicates significant difference as compared to the untreated control group (*P* < 0.05). **Indicates significant difference from UA treated alone (*P* < 0.05)

We further identified the role of IGFBP1, and the potential interactions between IGFBP1 and FOXO3. As expected, we showed that silencing of IGFBP1 overcame UA-inhibited cell growth in Bel-7402 and HepG2 cells (Fig. 6a). On the contrary, exogenous expressed IGFBP1 transfected into the cells showed to enhance UA-induced FOXO3a protein expression (Fig. 6b) and strengthened the UA-induced phosphorylation of p38 MAPK (Fig. 6c). More importantly, we further demonstrated that exogenous expression of IGFBP1 restored UA-inhibited growth in Bel-7402 and HepG2 cells in which endogenous IGFBP1 gene was previously silenced (Fig. 6d). Together, these findings suggested the potential interplay between the tumor suppressors IGFBP1 and FOXO3, and the feedback regulatory axis, resulting in reciprocal pathways that mediated the overall response of UA in HCC cells. These results also confirmed the crucial role of modulation of IGFBP1 gene expression in this process.

### In vivo anti-tumor efficacy of UA in subcutaneous HCC tumor-bearing nude mice model

**Fig. 7:**
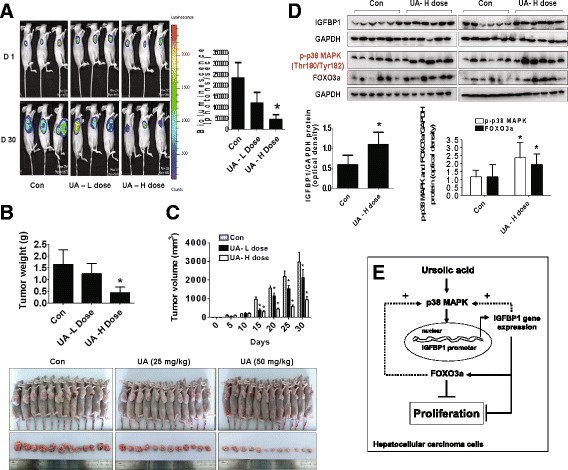
In vivo anti-tumor efficacy of UA in subcutaneous HCC tumor-bearing nude mice. Mice (n = 12/group) were divided to 3 groups [Con (saline), Low (L, 25 mg/kg) and High doses of UA (H, 50 mg/kg)], and UA was given daily around the 10^th^ day after tumor cells injection by gavages for up to 30 days. **a**, The xenografts were assessed by in vivobioluminescence imaging at the first and the end of the experiments [on day 1 (D 1) and Day 30 (D 30)]. The tumor growth was monitored by injecting luciferin in the mice followed by measuring bioluminescence using IVIS Imaging System. Imaging and quantification of signals were controlled by the acquisition and analysis software living image as described in the Materials and Methods section. Representative images are shown. **b** and **c**, The xenografts were harvested on day 30, and the volume and weight of tumors were measured. **d**, At the end of the experiments, xenografted tumors in each group were isolated and the tumors lysates were processed for detecting IGFBP1, FOXO3a protein and phosphorylation of p38 MAPK by Western blot. GAPDH was used as loading control. The bar graphs represented the tumor weight and volume of mice results as mean ± SD. *Indicates the significant difference from untreated control (*p* < 0.05). **e**, The diagram shows that UA inhibits growth of HCC cells through p38 MAPK-mediated induced expressions of IGFBP1 and FOXO3a. The interactions and correlations between IGFBP1 and FOXO3a, and the feedback regulatory loop of p38 MAPK by IGFBP1 and FOXO3a resulting in reciprocal pathways, contribute to the overall effects of UA

We also tested the effect of UA in HCC tumor growth in nude mouse xenografted cancer model. We found that, compared to the control, the UA-treated mice (50 mg/kg) showed a significant growth-inhibitory effect as assessed by the Xenogen IVIS200 System (Fig. 7a). In addition, we noticed a significant reduction of the tumor weight and sizes as compared to the control (Fig. 7b–c). By Western blot, fresh tumors harvested from the aforementioned experiment showed that high dose of UA (50 mg/kg) efficiently increased phosphorylation of p38 MAPK and protein expressions of IGFBP1 and FOXO3a as compared to that in the control group (Fig. 7d).

## Discussion

Chinese herbal medicines and its components have drawn a great attention for their potential impact in the treatment of many cancer types. Increasing numbers of studies demonstrated that ursolic acid, a pentacyclic triterpenoid found in medicinal herbs and fruits, inhibited the proliferation and induced the apoptosis in several types of cancers including HCC cells. We previously showed that UA inhibited growth and induced apoptosis of HepG2 HCC cells through AMPKα-mediated inhibition of Sp1; this in turn results in inhibition of DNA methyltransferase 1 [10]. In this study, we further explored the potential mechanism by which UA controls HCC cell growth. To this end, we showed that UA inhibited growth of HCC cells through p38 MAPK-mediated induction expression of transcription factor IGFBP1 and FOXO3a in reciprocal interacted fashion.

UA was found to inhibit growth in multiple HCC cell lines in the current study confirming the tumor suppressing properties of this agent in HCC cells. We did not observe the time-dependent effects of cell growth inhibition by UA and the reasons remained unclear. Whether longer time treatment (>72 h) showed significant changes needs to be determined. On the other hand, the possible desensitization of prolonged exposure of UA to the cells may also be responsible for this occurrence, which required to be confirmed. In our study, compared to the untreated control cells, there was actually 19 % more G0/G1 phase cell arrest by UA treatment. We believed that this may explain the significant inhibition of cell growth by UA, while the possible UA-induced apoptosis of HCC cells may also occurred, which required to be tested further. It was possible that the inhibition of proliferation could be in part a consequence of increased cell apoptosis. In addition, we demonstrated that UA inhibited growth of HCC cells through not only AMPKα but also p38 MAPK indicating that activation of these two signaling pathways may be required for UA induced HCC growth inhibition [10]. These pathways reported to be associated with the anti-cancer effects were also found in other studies suggesting the common signaling network that mediated the anti-tumor responses of UA [10, 41–43]. Moreover, regulation of FOXO3a and IGFBP1 through p38 MAPK signaling pathway haven been shown in other studies [44, 45]. Of note, inactivation of p38 was also reported to be involved in the FOXO3a activation and expression [46]. Thus, the true role of AMPKα and p38 MAPK signaling in modulating the FOXO3a and/or IGFBP1 expressions required to be determined.

Moreover, our data supported crucial function of IGFBP1 in the current study. Increasing evidence suggested an important role of IGFBP in the development and progression of several types of cancers [16–18, 47]. However, the expression and function of IGFBP1 in HCC development remains controversial, and little is known about its true role including diagnostic and prognostic values in HCC. In fact, paradoxical data have been reported in terms of the serum level and expression of IGFBP1 in patients with HCC [21, 22]. One study showed that that IGFBP1 may function as a tumor suppressor gene by blunting the IGF axis [21]. Consistent with this, our results implied that IGFBP1, acted as a tumor suppressor, could be a potential target in the HCC therapy.

We also demonstrated the involvement of FOXO3a that mediated the anti-HCC cell growth by UA. As a potential tumor suppressor, FOXO3a has been associated with many physiological and pathological processes, including proliferation, differentiation, cell cycle arrest, apoptosis and tumorigenesis [23, 24, 48, 49]. Our results implied that induction of FOXO3a was required in mediating the UA-inhibited HCC growth, which confirmed the tumor suppressor property of this transcription factor played in this process. This was the first report demonstrating the role of FOXO3a expression involving in the inhibition of HCC growth by UA, indicating that multiple potential targets may be involved in the anti-HCC effects of UA.

Furthermore, our data also implicated in the correlation between FOXO3a and IGFBP1, implying that IGFBP1 may be upstream of FOXO3a and mediated the positive feedback regulatory loop of p38 MAPK by FOXO3a. This contributed to the UA-inhibited HCC cell growth. The association between FOXO family and IGFBP1 has been shown in other studies [40, 50, 51]. For example, FOXO3a was found to bind to the IGFBP1 proximal promoter region and activated promoter activity thereby regulating its functions [40, 50]. Nevertheless, the true mechanism underlying this regulation still remain to be determined. Moreover, we demonstrated a novel feedback regulation of p38 MAPK by FOXO3a and IGFBP1, and this kinase regulatory loop may contribute to the overall inhibitory effects of UA on HCC cell growth. However, the other potential signaling pathways and up- or downstream mediators involving in this regulatory axis, and the true correlation between FOXO3a and IGFBP1 required to be elucidated in the future studies. Collectively, our findings indicated that targeting IGFBP1 and FOXO3a may be an alternative strategy in the treatment of HCC that warrants further investigation.

More importantly, our in vivo data were consistent with the findings from that in vitro, confirming the effect of UA on liver cancer growth inhibition and regulation of IGFBP1, FOXO3a expression, and p38 MAPK phosphorylation. The doses used for UA in the current study were similar to other reports demonstrating the significant effects in inhibiting growth of several cancer types including HCC [34–36]. Nevertheless, more experiments are needed to further elucidate the important role and the correlation between IGFBP1 and FOXO3a in this process using cells stable transfected with shRNAs or exogenous expressed IGFBP1 and/or FOXO3a genes in animal model.

## Conclusion

Overall, our results show that UA inhibits HCC cell proliferation through p38 MAPK-mediated induction of IGFBP1 gene expression and upregulation of FOXO3a. The inter-correlation between IGFBP1 and FOXO3a, and positive feedback regulatory loop of p38 MAPK by IGFBP1 and FOXO3a resulting in reciprocal pathways, contribute to the overall effects of UA (Fig. 7e). This in vitro and in vivo study corroborates a potential novel mechanism by which UA controls HCC cell growth and implies that the rational targeting IGFBP1 and FOXO3a can be potential for therapeutic strategies against HCC.

## Abbreviations

p38MAPK: P38 mitogen-activated protein kinase
DMSO: Dimethylsulfoxide
RT: Room temperature
qRT-PCR: Quantitative Real-Time PCR
MTT: 3-(4, 5-dimethylthiazol-2-yl)-2, 5-diphenyltetrazolium bromide
DMSO: Dimethyl sulfoxide
UA: Ursolic acid
HCC: Hepatocellular carcinoma
IGF: Insulin-like growth factor
IGFBP-1: Insulin-like growth factor binding protein 1
TCM: Traditional Chinese medicine
DNMT1: DNA (cytosine-5-)-methyltransferase 1
AMPKα: AMP-activated protein kinase alpha
GSK3β: Glycogen synthase kinase 3 beta
FoxO: Forkhead box class O
siRNAs: Small interfering RNAs
GLuc: Gaussia luciferase
SEAP: Secreted alkaline phosphatase
