# Erratum to: Inter-regulation of IGFBP1 and FOXO3a unveils novel mechanism in ursolic acid-inhibited growth of hepatocellular carcinoma cells

**DOI:** 10.1186/s13046-016-0343-x

**Published:** 2016-05-17

**Authors:** LiJun Yang, Qing Tang, Jingjing Wu, Yuqing Chen, Fang Zheng, Zhenhui Dai, Swei Sunny Hann

**Affiliations:** Laboratory of Tumor Biology and Target Therapy, The Second Clinical Medical Collage, University of Guangzhou Traditional Chinese Medicine, Guangzhou, Guangdong Province 510120 China; Department of Radiation Therapy, Guangdong Provincial Hospital of Chinese Medicine, The Second Clinical Medical Collage, University of Guangzhou Traditional Chinese Medicine, Guangzhou, Guangdong Province 510120 China; No. 55, Neihuan West Road, Higher Education Mega Center, Panyu District, Guangzhou, Guangdong Province 510006 PR China

## Erratum

Unfortunately, the original version of this article [1] contained two errors:

- The images published for Figs. 2, 3, 4, 5, 6 and 7 were not final and contained red labels with the authors’ corrections.
- The name of the first author in the author list was given as “Li Jun Yang” instead of “LiJun Yang”.

**Fig. 2:**
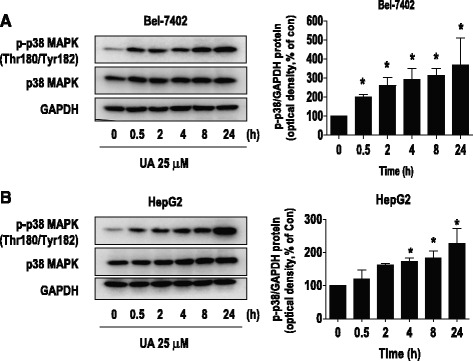
UA induced phosphorylation of p38 MAPK. **a**-**b**, Bel-7402 (**a**) and HepG2 (**b**) cells were exposed to UA (25 μM) for 24 h, followed by measuring the phosphorylation and protein expression of p38 MAPK by Western blot. The bar graphs represent the mean ± SD of p-p38 MAPK/GAPDH of three independent experiments. *Indicates significant difference as compared to the zero time group (*P* < 0.05)

**Fig. 3:**
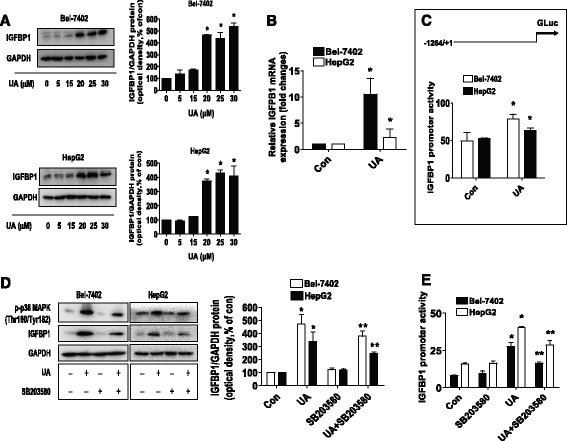
UA induced the protein, mRNA expression, and promoter activity of IGFBP1, which were blocked by SB203580. **a**-**b**, HepG2 and Bel-7402 cells were exposed to increased concentrations of UA or UA (25 μM) for 24 h. Afterwards, the expression of IGFBP1 protein (**a**) and mRNA (**b**) were detected by Western blot and qRT-PCR methods as described in the Materials and Methods section. *Indicates significant difference as compared to the untreated control group (*P* < 0.05) **c**, Bel-7402 and HepG2 cells were tranfected with wild type human IGFBP1 promoter reporter construct ligated to luciferase reporter gene and internal control secreted alkaline phosphatase (SEAP) for 24 h, followed by treating with UA (25 μM) for an additional 24 h. Afterwards, the IGFBP1 promoter activity were detected by the Secrete-Pair Dual Luminescence Assay Kit. **d**, HepG2 and Bel-7402 cells were treated with SB203580 (10 μM) for 2 h before exposure of the cells to UA (25 μM) for an additional 24 h. Afterwards, the expression of IGFBP1 protein and phosphorylation of p38 MAPK were detected by Western blot. The bars represent the mean ± SD of at least three independent experiments for each condition. *Indicates significant difference as compared to the untreated control group (*P* < 0.05); **Indicates significance of combination treatment as compared with UA alone (*P* < 0.05). **e**, Cellular protein was isolated from Bel-7402 and HepG2 cells cultured for 2 h in the presence or absence of SB203580 (10 μM) before transfection with control or above IGFBP1 constructs and exposing the cells to UA (25 μM) for an additional 24 h. Afterwards, the IGFBP1 promoter activity were detected by the Secrete-Pair Dual Luminescence Assay Kit. The bar graphs represent the mean ± SD of three independent experiments. *Indicates significant difference as compared to the untreated control group (*P* < 0.05); **Indicates significance of combination treatment as compared with UA alone (*P* < 0.05)

**Fig. 4:**
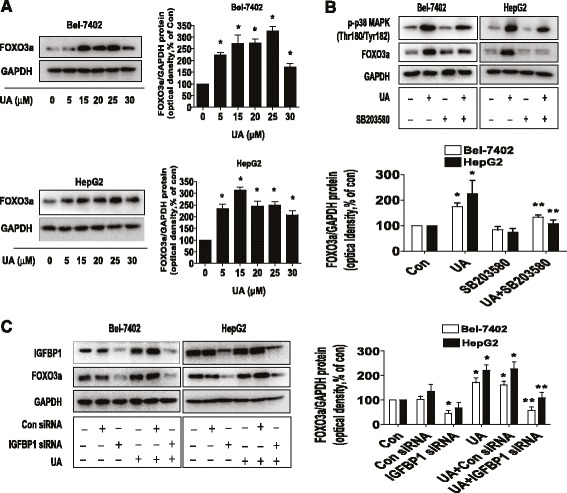
UA increased FOXO3a protein expression through activation of p38 MAPK and expression of IGFBP1. **a**, Bel-7402 and HepG2 cells were exposed to increased concentrations of UA for 24 h. Afterwards, the expression of FOXO3a protein was detected by Western blot. **b**, Bel-7402 and HepG2 cells were treated with SB203580 (10 μM) for 2 h before exposure of the cells to UA (25 μM) for an additional 24 h. Afterwards, the expression of FOXO3a protein and phoisphorylation of p38 MAPK were detected by Western blot. **c**, Bel-7402 and HepG2 cells were transfected with control or IGFBP1 siRNAs (50 nM each) for 24 h prior to exposure of the cells to UA (25 μM) for an additional 24 h. Afterwards, FOXO3a and IGFBP1 protein expressions were determined by Western blot, The bars represent the mean ± SD of at least three independent experiments for each condition. *Indicates significant difference as compared to the untreated control group (*P* < 0.05); **Indicates significance of combination treatment as compared with UA alone (*P* < 0.05)

**Fig. 5:**
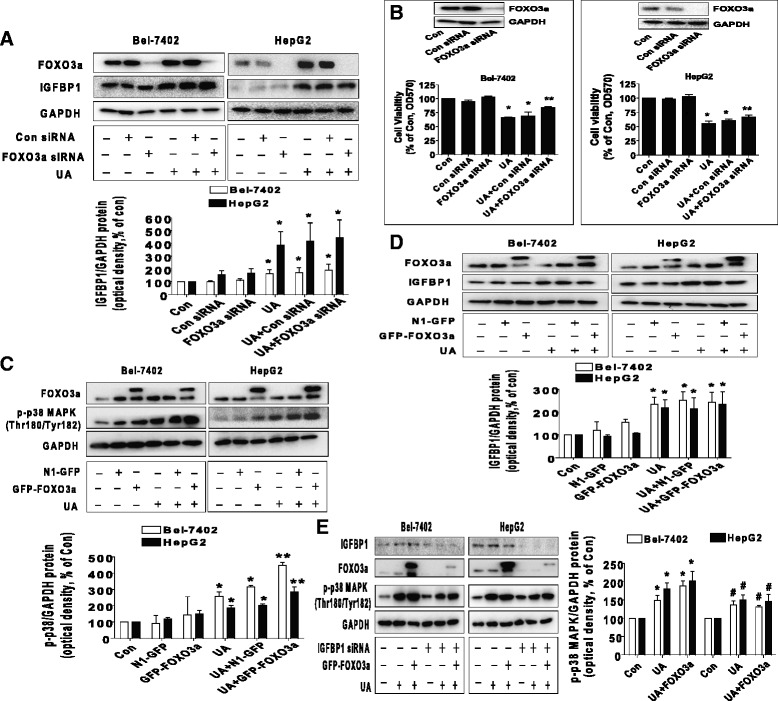
Silencing of FOXO3a overcame UA-induced cell growth inhibition and exogenous expressed FOXO3a enhanced UA-induced phosphorylation of p38 MAPK through IGFBP1. **a**, Bel-7402 and HepG2 cells were transfected with control and FOXO3a siRNAs for 24 h before exposing the cells to UA (25 μM) for an additional 24 h. Afterwards, FOXO3a and IGFBP1 protein expressions were determined by Western blot. **b**, Bel-7402 and HepG2 cells were transfected with control or FOXO3a siRNAs (up to 50 nM each) for 24 h prior to exposure of the cells to UA (25 μM) for an additional 24 h. Afterwards, FOXO3a protein expression and cell viability were determined by Western blot and MTT assays. Insert represents the protein expression of FOXO3a. **c**-**d**, Bel-7402 and HepG2 cells were transfected with control and FOXO3a overexpression vectors for 24 h before exposing the cells to UA (25 μM) for an additional 2 and 24 h, respectively. Afterwards, the protein levels of FOXO3a and p-p38 MAPK, and IGFBP1 protein expression were examined by Western blot. **e**, Bel-7402 and HepG2 cells silenced of IGFBP1 by siRNA previously were transfected with control and FOXO3a overexpression vector for 24 h before exposing the cells to UA (25 μM) for an additional 2 and 24 h, respectively. Afterwards, IGFBP1, FOXO3a protein and phosphorylation of p38 MAPK were determined by Western blot. Values in bar graphs were given as the mean ± SD from three independent experiments performed in triplicate. *Indicates significant difference as compared to the untreated control group (*P* < 0.05). **Indicates significant difference from UA treated alone (*P* < 0.01). ^#^Indicates significant difference as compared to the IGFBP1 siRNA alone group (*P* < 0.05)

**Fig. 6:**
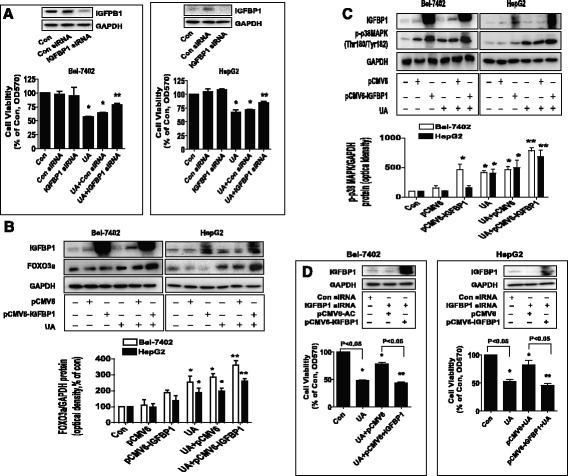
Overexpression of IGFBP1 enhanced the effect of UA on FOXO3a expression and phosphorylation of p38 MAPK, and restored UA-inhibited cell growth in cells silencing of endogenous IGFBP1 gene. **a**, Bel-7402 and HepG2 cells were transfected with control or IGFBP1 siRNAs (50 nM each) for 24 h prior to exposure of the cells to UA (25 μM) for an additional 24 h. Afterwards, IGFBP1 protein expression and cell viability were determined by Western blot and MTT assays. **b**-**c**, Bel-7402 and HepG2 cells were transfected with control and IGFBP1 overexpression vectors for 24 h before exposing the cells to UA (25 μM) for an additional 2 and 24 h, respectively. Afterwards, IGFBP1, FOXO3a protein levels and phosphorylation of p38 MAPK were determined by Western blot. **d**, Bel-7402 and HepG2 cells silenced of IGFBP1 by siRNA previously were transfected with control and IGFBP1 overexpression vectors for 24 h before exposing the cells to UA (25 μM) for an additional 24 h. Afterwards, IGFBP1 protein expressions and cell viability were determined by Western blot and MTT assays. Values in bar graphs were given as the mean ± SD from three independent experiments performed in triplicate. *Indicates significant difference as compared to the untreated control group (*P* < 0.05). **Indicates significant difference from UA treated alone (*P* < 0.05)

**Fig. 7:**
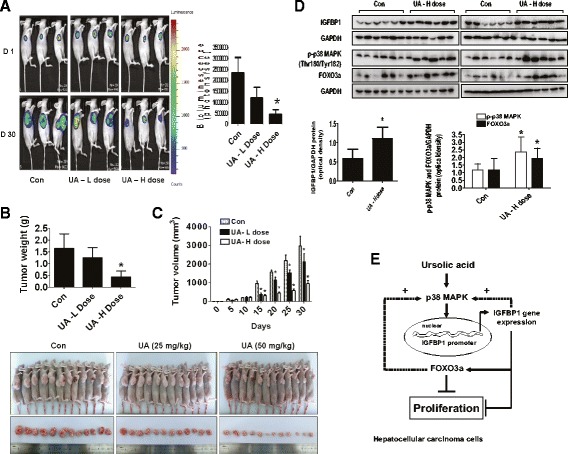
In vivo anti-tumor efficacy of UA in subcutaneous HCC tumor-bearing nude mice. Mice (n = 12/group) were divided to 3 groups [Con (saline), Low (L, 25 mg/kg) and High doses of UA (H, 50 mg/kg)], and UA was given daily around the 10^th^ day after tumor cells injection by gavages for up to 30 days. **a**, The xenografts were assessed by in vivo bioluminescence imaging at the first and the end of the experiments [on day 1 (D 1) and Day 30 (D 30)]. The tumor growth was monitored by injecting luciferin in the mice followed by measuring bioluminescence using IVIS Imaging System. Imaging and quantification of signals were controlled by the acquisition and analysis software living image as described in the Materials and Methods section. Representative images are shown. **b** and **c**, The xenografts were harvested on day 30, and the volume and weight of tumors were measured. **d**, At the end of the experiments, xenografted tumors in each group were isolated and the tumors lysates were processed for detecting IGFBP1, FOXO3a protein and phosphorylation of p38 MAPK by Western blot. GAPDH was used as loading control. The bar graphs represented the tumor weight and volume of mice results as mean ± SD. *Indicates the significant difference from untreated control (*p* < 0.05). **e**, The diagram shows that UA inhibits growth of HCC cells through p38 MAPK-mediated induced expressions of IGFBP1 and FOXO3a. The interactions and correlations between IGFBP1 and FOXO3a, and the feedback regulatory loop of p38 MAPK by IGFBP1 and FOXO3a resulting in reciprocal pathways, contribute to the overall effects of UA

The images and the name have been updated in the original article and are also correctly included in full in this erratum.
